# Birt–Hogg–Dubé syndrome: a case report and a review of the literature

**DOI:** 10.1080/20018525.2017.1292378

**Published:** 2017-02-20

**Authors:** Dea Kejlberg Jensen, Anders Villumsen, Anne-Bine Skytte, Mia Gebauer Madsen, Mette Sommerlund, Elisabeth Bendstrup

**Affiliations:** ^a^ Department of Respiratory Diseases and Allergy, Aarhus University Hospital, Aarhus, Denmark; ^b^ Institute of Clinical Medicine, Aarhus University, Aarhus, Denmark; ^c^ Department of Clinical Genetics, Aarhus University Hospital, Aarhus, Denmark; ^d^ Department of Urology, Aarhus University Hospital, Aarhus, Denmark; ^e^ Department of Dermatology, Aarhus University Hospital, Aarhus, Denmark

**Keywords:** Birt–Hogg–Dubé, fibrofolliculoma, pneumothorax, cysts, renal cancer, folliculin

## Abstract

**Background:** Birt-Hogg-Dubé syndrome (BHDS) is a rare autosomal dominant inherited syndrome caused by mutations in the folliculin coding gene (FLCN). The clinical manifestations of the syndrome involve the skin, lungs, and kidneys. Because of the rarity of the syndrome, guidelines for diagnosis and management of the patients with BHDS are lacking.

**Objective:** To present a case story and a review of the literature on BHDS in order to give an update on genetics, clinical manifestations, diagnosis, treatment, prognosis and follow-up strategies.

**Design:** Literature review and case story.

**Results:** A PubMed and Embase search identified 330 papers. BHDS is characterized by small benign tumors in the skin, spontaneous pneumothoraces caused by cysts in the lungs and a seven-fold increased risk of renal cancer. A case story of a young female patient presenting with pneumothorax and a family history of recurrent pneumothoraces in many relatives illustrates how the history and the diagnostic work up resulted in a diagnosis of BHDS.

**Conclusion:** BHDS is a rare inherited disorder. In patients with spontaneous pneumothorax or cystic lung disease without any obvious explanation, BHDS should be considered. Concomitant skin manifestations, a family history of familiar pneumothorax, renal cancers and skin manifestations supports the suspicion of BHDS. Early diagnosis is important in order to subject patients to systematic screening for renal cancers. A radiological surveillance strategy for renal cancer is proposed.

## Introduction

Birt–Hogg–Dubé syndrome (BHDS), also known as Hornstein–Knickenberg syndrome, is a rare, inherited syndrome known to involve the skin, lungs, and kidneys.[1] BHDS is an autosomal dominant monogenic disorder caused by constitutional mutations in the *FLCN* gene.[2–4] *FLCN* is a tumor suppressor gene,[5–9] and codes for the protein folliculin.[10–12] Clinical manifestations of the skin are fibrofolliculomas, trichodiscomas, and acrochordons, which primarily occur in the face, neck, and on the upper torso.[1,13] Lung cysts are the hallmark of the lung involvement, causing an increased risk of spontaneous pneumothorax.[14–17] The most severe manifestation of the syndrome is the predisposition to renal cell carcinoma (RCC).[16]

Until now, more than 600 families with BDHS have been described.[18] Due to its rarity, BHDS is unknown to many physicians. More families with BHDS may exist and the syndrome is likely to be under-diagnosed.

We present a case of BHDS and a review of the literature with focus on history, clinical manifestations, diagnosis, treatment, prognosis, and follow-up strategies and hope to draw attention to this rare inherited disorder.

## Case report

A 29-year old female was hospitalized in September 2014 with a spontaneous pneumothorax (SP) 2 days after completing a half marathon. The SP was treated successfully with drainage. The patient informed the treating physician that she knew of 11 other relatives with spontaneous pneumothorax, and therefore, she was referred for follow-up at the Department of Respiratory Diseases and Allergy at Aarhus University Hospital.

**Figure 1.**
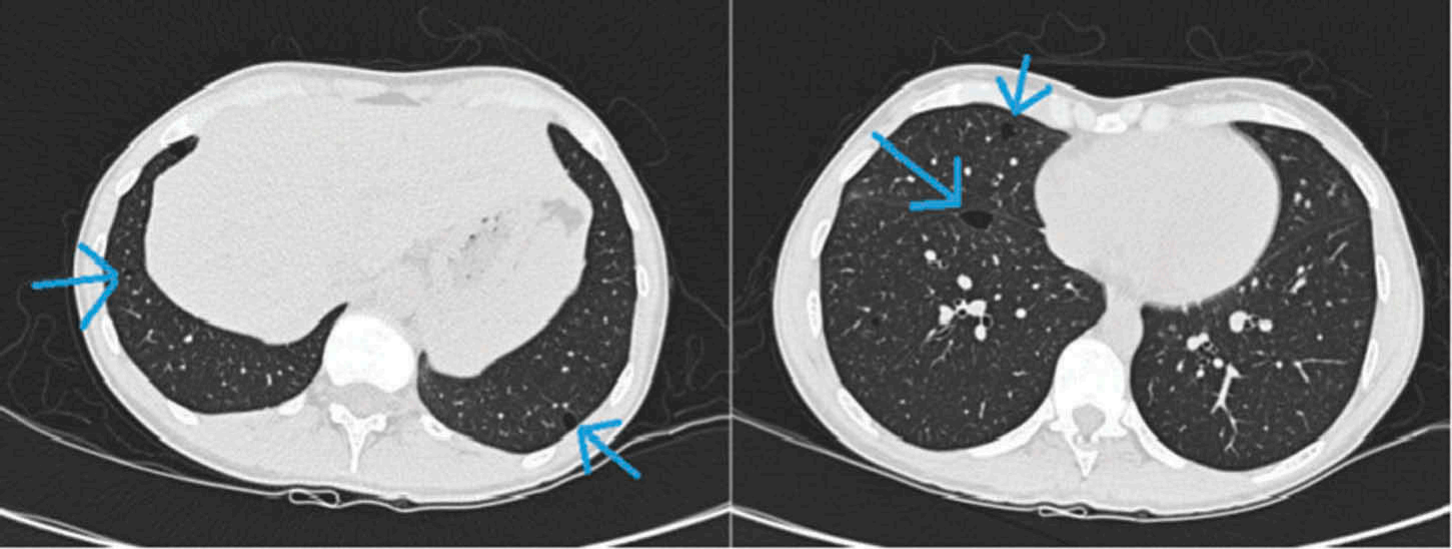
Lung cysts in a patient with Birt–Hogg–Dubé syndrome.

A high-resolution computed tomography (HRCT) scan showed multiple cysts in the basal parts of the lungs, which led to the suspicion of BHDS (Figure 1). Upon questioning, the patient described that her sister had fibrofolliculoma-like tumors in the face. Pulmonary function tests and magnetic resonance imaging (MRI) of the kidneys were normal.

Genetic screening revealed that the patient had a known pathogenic mutation in the *FLCN* gene, c.1285delC, which confirmed the diagnosis of BHDS.

**Figure 2.**
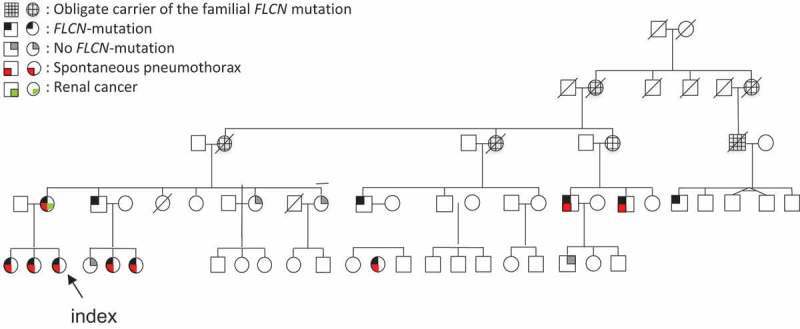
Family tree of the index patient with Birt–Hogg–Dubé syndrome.

Family members were offered genetic counseling and investigations. To date, 11 family members have been diagnosed with BHDS. A family tree is shown in Figure 2.

The patient and her affected family members were offered a follow-up program with MRI of the kidneys, and a pulmonary function test every second year.

## Methods

**Figure 3.**
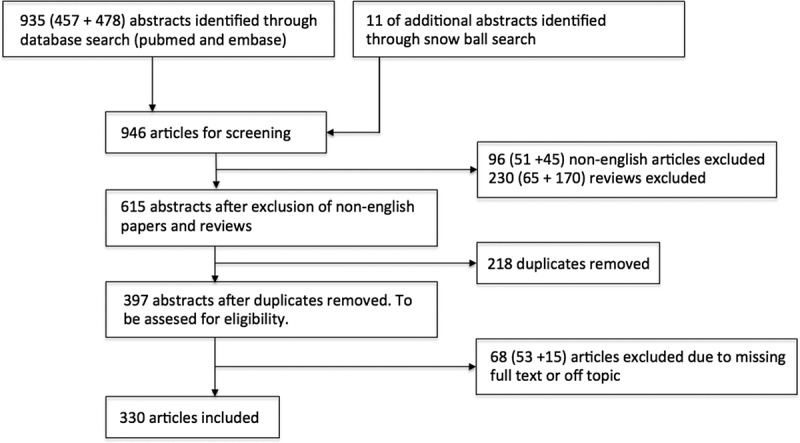
Search strategy.

A PubMed and Embase search with the terms ‘Birt–Hogg–Dubé syndrome’ and ‘Hornstein–Knickenberg syndrome’ was performed at 3 December 2016. Articles were restricted to those published in English language. Additional articles were identified by snowball search from reference lists of the already identified papers. In total 330 papers were identified. See Figure 3 for the search strategy.

## Results

### Background

BHDS is named after Arthur R. Birt, Georgina R. Hogg and James Dubé, who described the syndrome in 1977.[1] They reported a family of 70 members with 15 family members who developed fibrofolliculomas, trichodiscomas, and acrochordons on the scalp, forehead, face, neck, and upper torso after the age of 25. They also noticed that the skin changes were inherited in an autosomal dominant way. In 2005, Schmidt et al. [10] described the phenotype in 219 patients; see Table 1.

**Table 1.** The phenotype of 219 BHDS patients.[10]

Skin, lungs and kidneys	41.5%
Skin and lungs	41.5%
Skin	13%
Lungs and kidneys	2%
Skin and kidneys	2%

The autosomal dominant inheritance of the combination of skin manifestations and RCC in BHDS was first described by Toro et al. in 1999,[19] as a cohort of patients with renal tumors underwent an investigation for dermatological signs of BHDS. In this study, the overrepresentation of lung manifestations was also noticed.

In 2001, Schmidt et al. and Khoo et al located the gene locus of BHDS to be on the short arm of chromosome 17 [20,21] and in 2002 Nickerson et al linked the syndrome to the *FLCN* gene on chromosome 17, which encodes the protein folliculin.[2] Later, other mutations have been described.[22]

Zbar et al. [16] reported in 2002 that patients with BHDS had a sevenfold increased risk of developing RCC and a 50-fold risk of spontaneous pneumothorax.

In 2005, Schmidt et al. [10] noticed that the most commonly reported mutations in the *FLCN* gene resulted in premature termination and loss of function of folliculin. *FLCN* was therefore suggested to be a tumor suppressor gene.[23] Several FLCN interacting proteins including FNIP1 and FNIP2 have since been identified.[24,25] It has been suggested that FLCN is connected to numerous signaling pathways, including the energy-sensing mammalian target of rapamycin (mTOR) pathway. Whether the signaling is upregulated or inhibited is still being discussed.[26–32]

Since 2008, six BHDS symposiums have been held where researchers, clinicians, and patients meet and share information.[33] Currently, the BHDS foundation has knowledge of 616 BHDS families worldwide.[18]

### Lung manifestations

Multiple lung cysts are seen in approximately 67–90% of patients with BHDS and about 40% experience SP.[10,14,34,35] Compared to the background population, there is a 50-fold increased risk of SP.[16] About 40–75% will experience recurrent SP.[34,36] SP is mostly seen in adults, with a median age of 38 years at presentation, although a case of a seven-year-old boy with BHDS and pneumothorax has been reported.[36–38]

Neither gender predilection, association to smoking, nor other risk factors have been reported as predictors of the development of cysts or SP. Lung function is rarely affected.[34,36]

Lung cysts are diagnosed by a computed tomography (CT). The number of cysts is variable, ranging from 0 to 407 cysts.[39–41] The cysts are typically bilateral, located in the lower basal zones of the lungs, are of irregular shape, various size and have thin walls (Figure 1).[41–43] Repeated chest CTs after one year in five patients showed no development of the number or size of cysts.[44] Cyst size and basal localization correlate with an increased risk of SP.[14,34,36]

Differential diagnoses to BHD are other cystic lung diseases such as Langerhans cell histiocytosis, lymphangioleiomyomatosis (LAM), or other diseases with a high risk of secondary spontaneous pneumothorax, i.e. Marfan syndrome, chronic obstructive lung disease or emphysema.[39,45–47] It has been debated whether BHDS may contribute to the development of chronic obstructive pulmonary disease, but Cho et al. [48] found that this was not the case.

Most patients are asymptomatic and symptoms only appear when a SP is present. The treatment of SP in BHDS does not differ from the treatment of pneumothorax of other etiologies.[49] Almoosa et al. [50] found that chemical and surgical pleurodesis in patients with LAM decreased the pneumothorax recurrence rate, and pleurodesis after the first SP in BHDS has been suggested. Thorascopic pleural covering technique has also been suggested as a way of preventing recurrence of pneumothorax in patients with BHDS, but further studies are needed.[51,52]

### Skin manifestations

**Figure 4.**
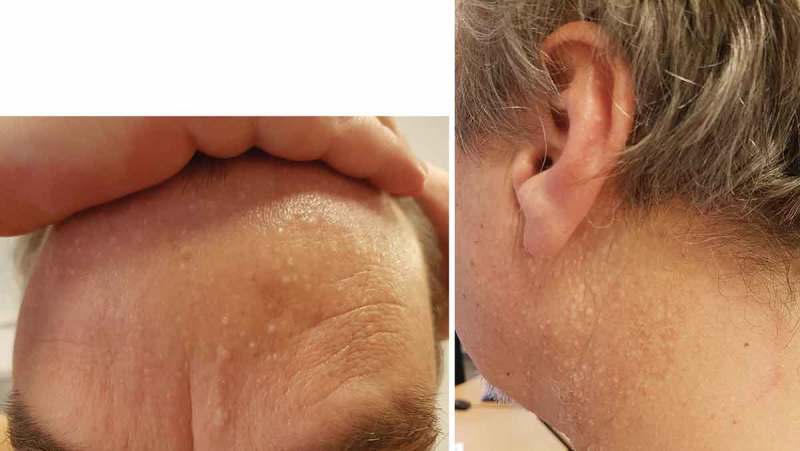
Fibrofolliculomas in a patient with Birt–Hogg–Dubé syndrome.

Skin manifestations are common in BHDS and are seen in approximately 58–90% of patients.[10,36,38,53,54] Most often, the tumors appear in the third or fourth decade and almost never before 25 years of age.[1,10,53] Fibrofolliculomas are the most frequent, but also trichodiscomas and acrochordons have been described.[1,34] Fibrofolliculomas present as multiple, pale yellow or white, slightly elevated, dome-shaped, and smooth tumors with a diameter of 2–4 mm (Figure 4). Fibrofolliculomas are predominantly located in the retroauricular area, face, neck, and upper torso and are macroscopically undistinguishable from trichodiscomas.[53] It is believed that fibrofolliculomas and trichodiscomas forms part of the same morphological spectrum.[55–57] These benign skin tumors offer an opportunity to diagnose patients with BHDS before lung cysts and/or renal tumors develop.

Acrochordons, also known as skin tags, are common skin lesions found in 25% of the general population and their presence is correlated to higher age and obesity.[58] They are small benign tumors often found in the armpit, neck, and groin and have no malignant potential. They are often seen in BHDS patients, but the relationship may be coincidental because the skin lesion is so common.

A diagnosis of fibrofolliculomas can be verified histologically by skin biopsies. In children and patients with no family history, the benign skin tumors may be the first symptom of BHDS. A histological confirmation of the fibrofolliculoma may therefore raise the suspicion of the rare syndrome of BHD. Differential diagnoses are sebaceous hyperplasia, and tumors such as fibroadenoma, basal cell carcinoma, and other syndromes with multiple benign tumors like Cowden, Rombo and Bazex-Dupré-Christol syndromes.[55]

Once the skin lesions have developed, they are permanent, and so far, no curative treatment is available. The skin lesions of BHDS are benign and are treated only for cosmetic reasons. Surgical and CO2 laser treatments can be used to remove the skin lesions, but the treatment is only temporary and the lesions often return over time.[53,57,59–61] A recent double-blind placebo-controlled randomized split-face study showed no effect of the topical mTOR inhibitor rapamycin on fibrofolliculoma in BHDS patients.[62]

### Kidney manifestations

Several studies have shown that patients with BHDS are at increased risk of renal cell tumors with varying malignancy potential.[16,19,63,64] Approximately 30% of the patients will develop renal tumors which corresponds to a seven-fold increased risk.[10,16,34–36,65] The risk increases with age, and BHDS patients older than 70 years of age have a relative risk of renal cell carcinomas (RCC) of 16%.[16,38]

RCC typically develop in middle-aged patients (mean age 50.7), although the earliest onset of renal cancer reported in BHDS patients was at 20 years of age.[54,65,66]

The histology of renal tumors in BHDS is different from sporadic renal tumors. Normally, 85% of renal tumors are of clear cell histology (ccRCC), 5–10% are papillary (pRCC), 5–10% chromophobe (chRCC) and 3–5% are oncocytomas (benign tumor).[64,67] In BHDS, 50% of the renal tumors are hybrid chRCC/oncocytoma and 33% are chRCC (both with a low malignant potential). The more aggressive ccRCC account for 9% and the benign oncocytomas account for 5%.[54,65,68]

BHDS patients may present with more than one tumor histology. 56% of the patients develop bilateral tumors and 65–77% develop multiple tumors ranging from one to 28 tumors with a mean of 5.3–7 tumors.[16,65,69] Renal cysts are common findings both in BHDS and in the general population, and it is not known whether the occurrence is more frequent in BHDS.[19,53]

The symptoms of RCC are often vague, and normally symptoms will not appear before the disease is advanced. Symptoms may be macroscopic hematuria, flank pain, fatigue, or a palpable tumor. If the disease is advanced, patients may have symptoms such as cough, bone pain, headache, anemia, and weight loss.[64] Therefore, all newly diagnosed patients should be offered abdominal imaging to exclude renal tumors. If no renal tumors are found at the time of diagnosis, regular screening should be offered.[6,70] Renal tumors may be diagnosed on ultrasonography, CT or MR imaging of the abdomen and confirmed by a biopsy.[64]

In patients with sporadic renal cancer without metastases, laparoscopic nephrectomy or partial nephrectomy are the standard treatment procedures, depending on the tumor size. In patients with small renal tumors (<4 cm), radiofrequency ablation (RFA) or cryoablation may be offered. In patients with metastatic ccRCC, chemotherapy are not effective, and therapy in this case is only palliative.[64] Systemic therapy in metastatic ccRCC are based on targeted and immune agents.

The national cancer institute (NCI) recommends that a nephron-sparing surgical technique should be used in order to preserve as much kidney function as possible.[71] Radical nephrectomy should only be performed if partial nephrectomy would result in an inferior oncologic outcome or a non-functioning kidney remnant.[65,72,75] This is due to the fact that BHDS patients are at risk of developing multiple and bilateral renal tumors. If radical nephrectomy is performed in these patients, they might develop tumors in the remaining kidney, and this may result in a more severe prognosis with renal insufficiency and dialysis. Therefore, it is recommended to postpone surgery until the largest solid tumor is 3 cm in diameter.[72,74]

A lifelong follow-up program with regular kidney scans, and nephron sparing surgery or ablative therapy of any tumors when they reach 3 cm, may prevent metastatic renal cancer and chronic renal insufficiency.[65,72] Toro et al. [75] suggested that BHDS patients without renal tumors should be followed up with an annual abdominal MR imaging. Others have suggested screening every 3–5 years.[57,75] Patients with renal tumors should be followed more closely.[65,72] It is suggested that patients with tumors < 1 cm are offered annual MRI and patients with tumors > 1 cm are evaluated more frequently depending on size, location, and growth rate.[72,75] Several studies have shown that small renal tumors are slow growing (approx. 0.3–0.8 cm year^–1^).[76,77]

The prognosis depends on tumor histology, size, and metastatic spread. Of BHDS kidney cancers 80–85% are slow growing with a low potential for metastasizing and a favorable prognosis. The ccRCCs are more aggressive and more likely to metastasize, which emphasizes the importance of regular scans to detect the tumors early.

### Diagnosis and follow-up

Early diagnosis of BHDS is paramount in order to include patients in RCC screening programs. Early diagnosis and treatment of RCC is important to prevent metastatic disease with a dismal prognosis.

Because the syndrome is rare, the diagnosis is often delayed for years. The variable presentation of the syndrome contributes to the diagnostic difficulties (Table 1).

Recently, the European BHDS consortium has proposed a set of criteria for the diagnosis of BHDS (Table 2).[70] Upon diagnosis of BHDS, the patients should undergo examination of the skin for fibrofolliculomas, CT imaging of the thorax for lung cysts, abdominal MR or CT imaging for renal tumors as well as genetic screening for pathogenic *FLCN* mutations.

**Table 2.** Diagnostic criteria for BHDS as proposed by the European BHDS consortium.[74]

A patient has Birt–Hogg–Dubé syndrome if:
The patient has a pathogenic FLCN mutation, *or*
The patient has > 4 fibrofolliculomas or trichodiscomas, at least one histologically confirmed, of adult onset, *or*
If 2 of the 3 following manifestations are present:
Multiple bilateral lung cysts with a basal predominance and no other apparent cause. With or without spontaneous pneumothorax.
A first-degree relative with BHDS
Early debut (< 50 years) of renal tumors or the presence of:
Multiple bilateral renal tumors
Renal tumors of the chromophobe/oncocytotic type

All BHDS patients should be offered genetic counseling by a clinical geneticist. If a pathogenic *FLCN* mutation is identified, all at-risk relatives should be offered genetic counseling and predictive testing. If the family meets the diagnostic criteria, but the genetic screening does not detect a causative mutation, all at-risk relatives should be offered genetic counseling and clinical evaluation.

Based on the information presented in this review, we propose a coordinator function, as shown in Table 3, to ensure that follow-up is offered to all BHDS patients.

**Table 3.** Proposed surveillance strategy following a diagnosis of BHDS.

Skin	No treatment or follow-up is needed
	Referral to dermatologist only for cosmetic reasons
Lungs	HRCT* and spirometry when BHDS is diagnosed
	No further follow-up necessary
Kidneys	All patients should be offered screening with abdominal MRI upon diagnosis**
	If no tumor is detected, abdominal MRI is recommended at the age of 25 and every second year thereafter
	If tumors are detected, the following procedure is suggested:
	Tumor size < 1 cm: patients are followed up with annual MRI
	Tumor size > 1 cm < 3 cm: patients are followed up with MRI every 6 months or offered ablative therapy
	Tumor size > 3 cm: nephron sparing surgery, or alternatively, ablative therapy (tumor < 4 cm) is recommended
	After renal surgery, MRI is performed each year for 5 years and every second year thereafter

*HRCT: high resolution computed tomography; MRI: magnetic resonance imaging

** First abdominal MRI at the age of 20.

#### Skin

The involvement of the skin is benign, and no follow-up is needed. However, patients with recurrent cosmetically disfiguring facial tumors can be referred to dermatologists for laser treatment.

#### Lungs

Progressive lung disease has not been reported, and in most cases, lung function is normal. The patients should know the symptoms of spontaneous pneumothorax and should be encouraged to seek medical help if they develop these symptoms. No systematic follow-up program is needed.

According to the British Thoracic Society guidelines, there are no specific life style precautions with respect to, for instance, air travelling, diving, strenuous exercise or wind instruments if a BHDS patient has had no pneumothorax.[76] However, a recent patient survey indicated an increased risk of pneumothorax after air travelling compared to the general population.[77] In families with BHDS, at-risk family members should be counseled and predictive genetic testing offered before diving.[77]

In patients with spontaneous pneumothorax or cystic lung disease without any obvious explanation, BHDS should be considered and the patient referred for genetic counselling. Concomitant skin manifestations or a family history of familiar pneumothorax, renal cancers and skin manifestations supports the suspicion of BHDS.

#### Kidneys

Due to the increased risk of metastatic renal cancer, follow-up and screening is important.

Although most BHDS patients develop slow-growing renal-tumor types, the more aggressive ccRCC and pRCC types have been described. No genotype-phenotype correlation has been reported. Therefore, the individual risk of aggressive renal tumors cannot be predicted. All patients with tumors should undergo the same follow-up program. After renal surgery, BHDS patients are still at risk of developing tumors, and should therefore continue the follow-up program. A proposed surveillance strategy for BHDS associated renal cancer is presented in Table 3.

## Conclusion

BHDS is a hereditary syndrome with an increased risk of fibrofolliculomas in the skin, multiple lung cysts predisposing to recurrent pneumothorax, and increased risk of renal cancer. Lung cysts are common, but they are usually asymptomatic unless a pneumothorax is present. The predisposition to RCC is the most feared complication, and it is important to diagnose and treat the patients before metastatic disease develops. The clinical expression of BHDS is variable. No genotype-phenotype correlations have been found, which makes early diagnosis and management of BHDS complex.

All BHDS patients should undergo regular abdominal imaging to detect renal tumors. Further research is needed to identify potential genotype–phenotype correlations and the exact pathogenesis in order to optimize the management of BHDS patients.
